# Venous thromboembolism and bleeding in cancer patients: role of inflammatory and cardiac biomarkers

**DOI:** 10.1093/eurheartj/ehaf1002

**Published:** 2025-12-12

**Authors:** Danielle Carole Roy, Tzu-Fei Wang, Ranjeeta Mallick, Dylan Burger, Marc Carrier, Philip Wells, Steven Hawken

**Affiliations:** School of Epidemiology and Public Health, Faculty of Medicine, University of Ottawa, 600 Peter Morand Crescent, Ottawa, ON, Canada K1G 5Z3; School of Epidemiology and Public Health, Faculty of Medicine, University of Ottawa, 600 Peter Morand Crescent, Ottawa, ON, Canada K1G 5Z3; Department of Medicine, University of Ottawa, Ottawa, ON, Canada; The Ottawa Hospital Research Institute, The Ottawa Hospital, Ottawa, ON, Canada; School of Epidemiology and Public Health, Faculty of Medicine, University of Ottawa, 600 Peter Morand Crescent, Ottawa, ON, Canada K1G 5Z3; The Ottawa Hospital Research Institute, The Ottawa Hospital, Ottawa, ON, Canada; Department of Medicine, University of Ottawa, Ottawa, ON, Canada; The Ottawa Hospital Research Institute, The Ottawa Hospital, Ottawa, ON, Canada; School of Epidemiology and Public Health, Faculty of Medicine, University of Ottawa, 600 Peter Morand Crescent, Ottawa, ON, Canada K1G 5Z3; Department of Medicine, University of Ottawa, Ottawa, ON, Canada; The Ottawa Hospital Research Institute, The Ottawa Hospital, Ottawa, ON, Canada; School of Epidemiology and Public Health, Faculty of Medicine, University of Ottawa, 600 Peter Morand Crescent, Ottawa, ON, Canada K1G 5Z3; Department of Medicine, University of Ottawa, Ottawa, ON, Canada; The Ottawa Hospital Research Institute, The Ottawa Hospital, Ottawa, ON, Canada; School of Epidemiology and Public Health, Faculty of Medicine, University of Ottawa, 600 Peter Morand Crescent, Ottawa, ON, Canada K1G 5Z3; The Ottawa Hospital Research Institute, The Ottawa Hospital, Ottawa, ON, Canada

**Keywords:** Venous thromboembolism, Bleeding, Biomarkers, Cancer, Inflammation

## Abstract

**Background and Aims:**

Patients with cancer have increased risk of venous thromboembolism (VTE) and bleeding. Inflammatory and cardiac biomarkers may predict these complications, but their role remains unclear. This study examined associations between two inflammatory-related markers (C-reactive protein and growth differentiation factor-15) and two cardiac markers [N-terminal pro-B-type natriuretic peptide and high-sensitivity troponin T (hs-TnT)] with VTE and clinically relevant bleeding in cancer patients.

**Methods:**

A *post hoc* analysis of the AVERT trial, which evaluated apixaban for VTE prevention in ambulatory cancer patients with a Khorana score of ≥2, was performed. Biomarkers were measured at baseline and 1 month, with C-reactive protein also at 3 months. Fine and Gray regression, accounting for competing risk of death and adjusted for age and advanced cancer, estimated subdistribution hazard ratios (SHRs) for VTE and clinically relevant bleeding.

**Results:**

Of 574 patients, 514 provided baseline samples. One- and 3-month samples were available from 454 and 447, and 378 and 364, patients without prior VTE and bleeding events, respectively. Elevated baseline growth differentiation factor-15 was associated with increased VTE risk [SHR 1.36, 95% confidence interval (CI) 1.01–1.84]. N-terminal pro-B-type natriuretic peptide (SHR 1.44, 95% CI 1.08–1.92) and C-reactive protein (SHR 1.38, 95% CI 1.07–1.76) were linked to bleeding risk. Increasing high-sensitivity troponin T from baseline to 1 month was associated with higher VTE risk (SHR 1.89, 95% CI 1.14–3.16). Nomograms were developed to estimate VTE and clinically relevant bleeding risks.

**Conclusions:**

Select inflammatory-related and cardiac markers were associated with VTE and bleeding risks in cancer patients, which can be determined using developed nomograms. Prospective research is needed to confirm these findings.

## Structured Graphical Abstract

**Structured Graphical Abstract:**
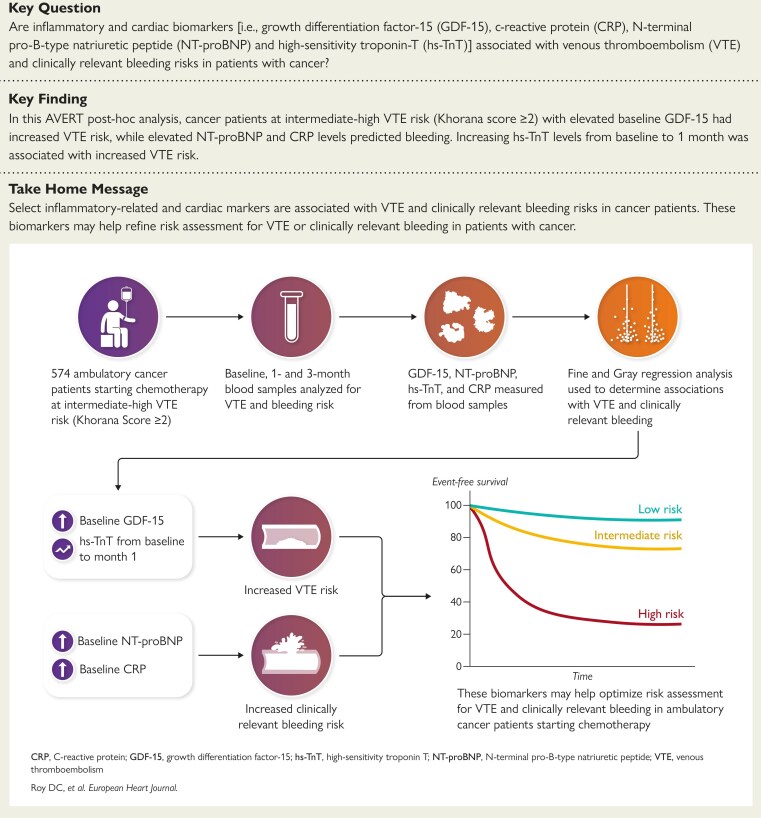

**See the editorial comment for this article ‘Primary prophylaxis of venous thromboembolism in ambulatory patients with active cancer’, by D. Farmakis**  ***et al*****., https://doi.org/10.1093/eurheartj/ehaf1090.**

## Introduction

Several risk factors and pathways contribute to a high risk of venous thromboembolism (VTE) in cancer patients. Concerning the aetiology, evidence supports inflammation and coagulation as interrelated processes. Briefly, inflammation promotes coagulation through inflammation-induced coagulation activation, inflammation-induced downregulation of natural anticoagulants, and inhibition of fibrinolysis.^1^ This link is supported by increased VTE risk in inflammatory conditions such as rheumatic arthritis,^2^ systemic lupus erythematosus,^2,3^ systemic sclerosis,^2,4^ and inflammatory bowel disease.^5,6^ This connection between inflammation and coagulation has prompted several studies to evaluate the relationship between inflammatory biomarkers and VTE risk. In two systematic reviews, higher levels of C-reactive protein were associated with an increased risk of VTE,^7,8^ and additional evidence indicated that C-reactive protein is a predictor of cardiovascular events and may aid in primary prevention of atherosclerotic cardiovascular disease.^9,10^ Other inflammatory-related markers, such as growth differentiation factor-15 (GDF-15) and interleukin-6 (IL-6), have also been linked to VTE.^11,12^ However, the association between GDF-15 and VTE in cancer patients remains a matter of debate. In our previous studies evaluating the risk of VTE and bleeding in ambulatory cancer patients with high GDF-15 at 1-month post-chemotherapy, increased GDF-15 levels were associated with an increased risk of both VTE and bleeding risk.^13,14^ However, in the prospective Vienna Cancer and Thrombosis Study (CATS) cohort, elevated GDF-15 was not linked to increased VTE risk, although high GDF-15 was related to bleeding risk.^15,16^

Cardiac biomarkers such as natriuretic peptides and troponins have also been implicated in VTE risk. Elevated levels of N-terminal pro-B-type natriuretic peptide (NT-proBNP) and high-sensitivity troponin T (hs-TnT) are associated with increased thrombotic risk in the general population and with stroke and bleeding in patients with atrial fibrillation.^17–21^ N-terminal pro-B-type natriuretic peptide is a marker of myocardial stress, and hs-TnT is a marker of myocardial damage.^22–24^ Although the exact mechanism remains unclear, it is hypothesized that NT-proBNP and hs-TnT may reflect a prothrombotic state since they are typically elevated in a variety of cardiac conditions,^17,22–24^ which are known to provoke VTE such as heart failure and peripheral atherosclerotic diseases.^25,26^ However, little is known about the relationship between cardiac biomarkers such as NT-proBNP, hs-TnT, and VTE risk in cancer patients.

In patients with cancer, a proinflammatory and prothrombotic state is exacerbated by malignancy itself and its treatment. However, the relationship between inflammatory-related and cardiac markers and VTE among patients with cancer remains less clear. While some studies have identified C-reactive protein as a VTE predictor in cancer patients,^27,28^ others, after adjusting for confounders, have not confirmed this.^29^ Interestingly, a recent study exploring the early dynamics of C-reactive protein levels in patients treated with immune checkpoint inhibitors found that a two-fold rise in C-reactive protein levels was associated with increased VTE risk.^30^

To address these knowledge gaps, this study investigated the association between baseline levels of two inflammatory-related markers (C-reactive protein and GDF-15), with GDF-15 more accurately reflecting stress responses than classic inflammation, and two cardiac markers (NT-proBNP and hs-TnT) with VTE in cancer patients at intermediate to high risk of VTE (defined as a Khorana score of ≥2). Following recent recommendations to assess biomarker changes over time,^31^ we also examined the relationship between longitudinal changes in these biomarkers and their relationship with VTE. In addition, we assessed both baseline and longitudinal trends in these biomarkers in relation to bleeding events.

## Methods

### Study design

This is a *post hoc* analysis of the AVERT trial (Apixaban to Prevent VTE in Patients with Cancer), evaluating the association between baseline and longitudinal biomarker levels for the prediction of cancer-associated VTE and bleeding. The AVERT trial was a randomized, double-blind placebo-controlled trial assessing the efficacy and safety of apixaban compared with placebo in preventing VTE in ambulatory cancer patients with intermediate to high risk of VTE (defined as a Khorana score of ≥2) between February 2014 and April 2018 from 13 Canadian hospitals. Patients were randomized 1:1 to apixaban 2.5 mg twice daily vs placebo for 6 months. Full details on the methodology and eligibility for the trial have been published.^32^

### Participants

The AVERT trial enrolled ambulatory cancer patients, aged 18 years or older, with newly diagnosed or progressive cancer and a Khorana score of ≥2. Eligible patients were initiating a new course of chemotherapy and provided written informed consent. At the time of inclusion, participants were also asked for consent to store blood samples for future secondary research use. In this study, we included all patients enrolled in the AVERT trial who consented for additional blood draw for research. Patients with missing blood samples at baseline or during follow-up were excluded.

### Variables and outcomes

Patient demographics and characteristics, including ethnicity, age, cancer type, cancer stage (defined as advanced if the patient had haematological cancer or Stage 3 or 4 solid cancer), sex (male or female as assigned at birth), and treatment group (i.e. apixaban vs placebo), were collected at the time of randomization. For this study, the primary outcome was occurrence of VTE, defined as objectively confirmed symptomatic or incidentally detected thromboembolic events, including proximal or distal lower or upper limb deep vein thrombosis (DVT), pulmonary embolism (PE), and splanchnic or cerebral vein thrombosis occurring within 7 months following enrolment. The secondary outcome was the composite of major bleeding and clinically relevant non-major bleeding (CRNMB) (both defined using the International Society on Thrombosis and Haemostasis definitions)^33,34^ within 7 months following enrolment. These outcomes differ from the original trial, which focused on proximal DVT, PE, and bleeding events within a 6-month follow-up period. However, all thrombotic and bleeding events were adjudicated by a blinded outcome adjudication committee for the full 7-month follow-up, allowing for inclusion of these outcomes in this *post hoc* analysis.

During the AVERT trial, blood samples were collected at baseline and at Months 1, 3, 6, and 7 following enrolment. In this study, we analysed blood samples and focused on biomarkers measured at baseline, Month 1, and Month 3. Particularly, we measured GDF-15, NT-proBNP, hs-TnT, and C-reactive protein levels at baseline and C-reactive protein levels at Months 1 and 3. In addition, we included previously measured biomarkers at Month 1 including GDF-15, NT-proBNP, and hs-TnT levels, which were part of previous research.^13^ C-reactive protein was the only biomarker tested at Month 3 given previous evidence suggesting an association between C-reactive protein changes in the first 3 months and VTE risk in cancer patients initiating immune checkpoint inhibitor therapy.^30^ Due to funding and project constraints, the biomarkers were measured at three different labs. C-reactive protein was measured using particle-enhanced immunoturbidimetric assays on the Roche cobas 6000 automated chemistry analyser (at baseline) or Roche c702 instrument (Months 1 and 3). Growth differentiation factor-15 was measured using an ELISA immunoassay (Biotechne Quantikine GDF-15, R&D systems) on the Roche cobas 6000 automated chemistry analyser (baseline) and an electrochemiluminescence immunoassay (Roche Elecsys assay, Roche Diagnostics) on a cobas e411 platform (Month 1). N-terminal pro-B-type natriuretic peptide and hs-TnT were measured using electrochemiluminescence immunoassays on the Roche cobas 6000 automated chemistry analyser (baseline) or Roche cobas e411 platform (Roche Diagnostics, Mannheim, Germany) (Month 1).

### Statistical analysis

We assessed patient characteristics and biomarker levels between patients with and without VTE and bleeding using descriptive statistics. Since all biomarker levels were right skewed, we reported biomarker levels as medians and interquartile ranges. Prior to imputation and regression analyses, biomarkers were log transformed and standardized by subtracting the mean and dividing by the standard deviation (SD). Participants with partial missing biomarker data were retained, and missing biomarker levels were imputed using single imputation predictive mean matching. Participants with fully missing biomarker data at baseline, Month 1, or Month 3 were excluded from analyses, as these may have reflected withdrawal of consent. The analytical sample included all participants with at least one baseline biomarker measurement (*n* = 514). While the sample size was fixed, the number of events per variable was adequate to permit reliable estimation of Fine and Gray subdistribution hazard models and ensure robust model performance.

To assess the association between biomarkers and clinical outcomes, we used Cox proportional hazards and Fine and Gray competing risk regression models. The Cox regression models were used to detect departures from linearity for continuous biomarkers. Specifically, to examine potential non-linear relationships between biomarkers and outcomes, we compared the Akaike information criterion (AIC) of restricted cubic spline Cox regression models with four knots (located at the 5th, 35th, 65th, and 95th percentiles) and three knots (located at the 10th, 50th, and 95th percentiles) with the AIC of a linear model. The model with the smallest AIC was considered to be having the best fit. This approach is recommended and well described by Frank Harrell^35^ in *Regression Modeling Strategies*. We also plotted the restricted cubic spline models with four knots to visually assess for non-linearity. To verify proportionality over time, scaled Schoenfeld residuals were plotted over time and visually inspected for non-proportionality. In cases where biomarkers showed evidence of non-linear associations with outcomes, we plotted the hazard ratio across different biomarker levels. For biomarkers demonstrating a linear relationship with VTE or bleeding, we used Fine and Gray competing risk regression models, incorporating linear terms and adjusting for the competing risk of all-cause mortality. To confirm the proportional subdistribution hazards assumption was met, we plotted the cumulative incidence functions and visually inspected for consistent, non-crossing separation between the groups.

We performed separate regression analyses to evaluate how baseline biomarker levels and changes in biomarkers over time (from baseline to Month 1 or Month 3) were associated with the risk of future VTE and bleeding. To assess the baseline association, only the transformed and standardized baseline biomarker values were included in the model. For changes over time, we included the relative differences in log biomarker levels (i.e. log Month 1 or log Month 3 values minus log baseline) while adjusting for the log baseline value. Multivariable models were used to account for potential confounders, specifically age and metastatic/advanced cancer. These confounders were selected *a priori* based on a causal directed acyclic graph. To help with clinical applicability, we generated nomograms to help describe the association between biomarkers and outcomes. Sensitivity analyses were performed to assess the robustness of results, one analysis without imputations, another stratified by an intervention group (i.e. apixaban vs placebo), a third adjusting for different non-chemotherapy cancer therapies (i.e. targeted therapy and radiation therapy) administered at the time of biomarker measurement, and a fourth adjusting for apixaban. We additionally evaluated the effect of apixaban on biomarker levels by performing a multiplicative interaction analysis including an interaction term between apixaban and biomarker levels for both outcomes. No additive interaction analyses were performed. Biomarkers were assessed for their association with VTE in the placebo group and bleeding in the apixaban group, as these populations were considered higher risk for these outcomes. For comparison, we also analysed the risk of VTE and bleeding in previously defined C-reactive protein change subgroups including C-reactive protein rise (defined as a two-fold or greater increase in C-reactive protein levels from baseline) and C-reactive protein decline (defined as a 50% or greater decrease in C-reactive protein levels from baseline)^30^ using the values from Month 1 to Month 3. In addition, we evaluated whether the addition of biomarkers improved the performance of existing clinical scores and performed a stratified analysis by the Khorana score for baseline biomarkers significantly associated with VTE risk. For all analyses, a *P*-value of <.05 was considered statistically significant, and all analyses were performed in SAS version 9.4 (SAS Institute, Inc., Cary, NC, USA) or RStudio (R Core Team, 2024, Vienna, Austria).

## Results

**Figure 1:**
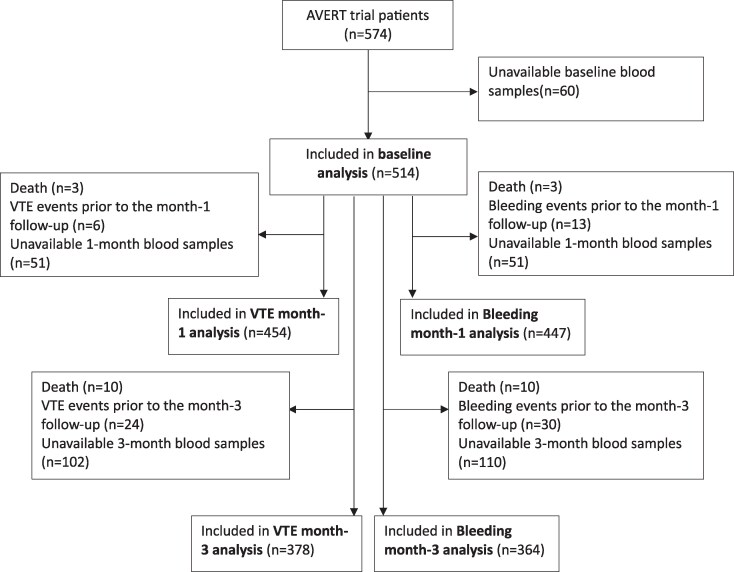
Flow diagram of patients included in the different venous thromboembolism and clinically relevant bleeding analyses

Of 574 patients enrolled in the AVERT trial between February 2014 and April 2018, a total of 514 patients consented to providing blood samples at baseline (*Figure 1*). Of these, 43 patients developed VTE events (24 DVT, 15 PE, 2 combined DVT and PE, and 2 splanchnic vein thrombosis), 46 developed bleeding events (11 major bleeding and 35 clinically relevant non-major bleeding events), and 53 (9.7%) patients died during the 7-month follow-up. To analyse the change in biomarker levels from baseline to Month 1, we excluded patients who did not consent to providing 1-month blood samples as well as those who died or developed an event prior to the 1-month follow-up visit (*Figure 1*). In the VTE and bleeding 1-month analyses, there were 37 and 38 competing events, respectively. Similarly, to analyse the change in C-reactive protein levels from baseline to Month 3, we also excluded patients who did not provide consent and those with previous events or deaths (*Figure 1*). In the VTE and bleeding 3-month analyses, there were 22 and 24 competing events, respectively. At baseline, 28 GDF-15, 31 NT-proBNP, 56 hs-TnT, and 17 C-reactive protein values were missing and therefore imputed. At Month 1, 12 GDF-15, 12 NT-proBNP, 8 hs-TnT, and 6 C-reactive protein values required imputation. At Month 3, no values were imputed. Overall, 21.0% of participants had at least one missing predictor in the baseline regression models used.

Overall, for the 514 patients included at baseline, the mean (SD) age at enrolment was 62 (12.1) and the median follow-up was 196 (interquartile range: 183–204) days. A total of 3.7% of participants (*n* = 19) were censored prior to 6 months due to loss to follow-up or withdrawal, while the majority of remaining censored observations occurred towards the end of the study, between 6 and 7 months of follow-up. The most common primary cancer sites were gynaecologic (*n* = 138), lymphoma (*n* = 131), and pancreatic (*n* = 67). Among the patients, 41.8% were male and 50.2% patients were randomized to receive apixaban. Consistent with the main findings of the AVERT trial, the incidence of VTE was higher among patients randomized to placebo than those randomized to apixaban (69.8% vs 30.2%) (*Table 1*). The mean (SD) biomarker levels stratified by VTE and bleeding and the standardized mean differences (SMDs) are presented in *Table 1*.

**Table 1:** Baseline characteristics and laboratory results of cancer patients with and without venous thromboembolism

	Venous thromboembolism (*n* = 43)	No venous thromboembolism (*n* = 471)	Standardized mean difference (95% confidence interval)	Bleed (*n* = 46)	No bleed (*n* = 468)	Standardized mean difference (95% confidence interval)	Missing data, *n* (%)
Age (years), mean (standard deviation)	64.0 (11.1)	62.0 (12.1)	0.11 (−0.21–0.42)	62.5 (14.7)	62.0 (11.8)	−0.06 (−0.37–0.24)	0
Male sex, *n* (%)	20 (46.5)	195 (41.4)		15 (32.6)	200 (47.7)		0
Body mass index (kg/m^2^), median (interquartile range)	27.5 (25.1–32.0)	27.7 (23.7–35.1)	−0.003 (−0.31–0.31)	27.7 (23.9–33.5)	27.7 (23.7–35.1)	0.06 (−0.25–0.36)	0
Previous venous thromboembolism, *n* (%)	3 (7.0)	13 (2.8)		2 (4.4)	14 (3.0)		0
Randomized to apixaban, *n* (%)	13 (30.2)	245 (52.0)		30 (65.2)	228 (48.7)		0
Randomized to placebo, *n* (%)	30 (69.8)	226 (48.0)		16 (34.8)	240 (51.3)		0
Antiplatelet use, *n* (%)	7 (16.3)	100 (21.2)		10 (21.7)	97 (20.7)		0
Known metastatic or advanced cancer, *n* (%)	35 (81.4)	418 (88.8)		44 (95.7)	409 (87.4)		43 (8.4)
Primary cancer site, *n* (%)							
Brain	3 (7.0)	19 (4.0)		1 (2.2)	21 (4.5)		0
Breast	0	17 (3.6)		1 (2.2)	16 (3.4)		0
Gastrointestinal	9 (20.9)	50 (10.6)		5 (10.9)	54 (11.5)		0
Gynaecologic	8 (18.6)	130 (27.6)		17 (37.0)	121 (25.9)		0
Haematological	0	17 (3.6)		1 (2.2)	16 (3.4)		0
Lung	0	51 (10.8)		2 (4.3)	49 (10.5)		0
Lymphoma	8 (18.6)	123 (26.1)		12 (26.1)	119 (25.4)		0
Pancreas	12 (27.9)	55 (11.7)		6 (13.0)	61 (13.0)		0
Other	3 (7.0)	9 (1.9)		1 (2.2)	11 (2.4)		0
Laboratory test results at baseline, mean (standard deviation)							
log(growth differentiation factor-15) (pg/mL)	7.3 (0.7)	7.1 (0.7)	0.27 (−0.04–0.59)	7.1 (0.8)	7.1 (0.7)	0.09 (−0.22–0.39)	28 (5.5)
log(N-terminal pro-B-type natriuretic peptide) (pg/mL)	4.8 (1.0)	4.9 (1.0)	−0.04 (−0.35–0.28)	5.2 (1.2)	4.8 (1.0)	0.39 (0.08–−0.69)	31 (6.0)
log(high-sensitivity troponin T) (pg/mL)	2.2 (0.7)	2.1 (0.5)	0.22 (−0.09–0.54)	2.1 (0.5)	2.2 (0.5)	−0.04 (−0.34–0.27)	56 (10.9)
log(C-reactive protein) (mg/dL)	0.2 (1.4)	0.01 (1.3)	0.12 (−0.19–0.43)	0.5 (1.3)	−0.02 (1.3)	0.41 (0.11–0.71)	17 (3.3)
Laboratory test results at Month 1, mean (standard deviation)^a^							
log(growth differentiation factor-15) (pg/mL)	8.0 (0.8)	7.6 (0.8)	0.41 (−0.04–0.79)	7.8 (1.0)	7.6 (0.8)	0.21 (−0.16–0.58)	12 (2.6)
log(N-terminal pro-B-type natriuretic peptide) (pg/mL)	5.1 (1.0)	4.7 (1.3)	.30 (−0.07–0.67)	5.1 (1.1)	4.7 (1.3)	0.34 (−0.03–0.72)	12 (2.6)
log(high-sensitivity troponin T) (pg/mL)	2.3 (0.8)	1.9 (1.0)	.49 (−0.12–0.86)	1.9 (.8)	1.9 (1.0)	0.04 (−0.33–0.41)	8 (1.8)
log(C-reactive protein) (mg/dL)	−0.7 (1.5)	−0.9 (1.4)	.16 (−0.21–0.53)	−0.6 (1.6)	−0.9 (1.4)	0.18 (−0.19–0.55)	6 (1.3)
Laboratory test results at Month 3, mean (standard deviation)^a^							
log(C-reactive protein) (mg/dL)	−0.2 (1.5)	−1.2 (1.4)	0.67 (−0.13–1.21)	−1.6 (1.5)	−1.1 (1.4)	0.38 (−0.88–0.13)	0

^a^At Month 1, 30 patients had venous thromboembolism and 424 patients had no venous thromboembolism, and at Month 3, 16 patients had venous thromboembolism and 362 patients had no venous thromboembolism.

At baseline, there was no evidence of non-linear associations between any of the four biomarkers and outcomes. Therefore, we used Fine and Gray competing risk regression with linear terms to determine the association between baseline biomarkers and future risk of VTE and bleeding, with death as a competing risk (*Tables 2* and *3*). The results suggest that elevated baseline GDF-15 was associated with an increased risk of VTE [subdistribution hazard ratio (SHR) 1.36, 95% confidence interval (CI) 1.01–1.84], but not bleeding, after adjustment for age and advanced cancer (*Table 3*). Biomarkers that were not associated with VTE but showed a positive association with increased risk of bleeding included NT-proBNP and C-reactive protein [SHR 1.44 (1.08–1.92); SHR per 1 SD log increase: 1.38 (1.07–1.76), respectively] (*Table 3*) after multivariable adjustment. In our sensitivity analyses excluding imputed data, adjusting for non-chemotherapy cancer therapies, adjusting for apixaban, and focusing on patients randomized to placebo or apixaban, similar hazard ratios were reported (*Table 4*; Supplementary data online, *Tables S1*–*S5*).

**Table 2:** Unadjusted association between biomarkers and risk of venous thromboembolism and clinically relevant bleeding in cancer patients from the AVERT trial

Predictor^a^	Venous thromboembolism			Bleeding		
	*N* of events	Subdistribution hazard ratio (95% confidence interval)^b^	*P*-value	*N* of events	Subdistribution hazard ratio (95% confidence interval)^b^	*P*-value
Growth differentiation factor-15						
Baseline	43	1.30 (0.98–1.73)	.068	46	1.10 (0.80–1.51)	.58
Change from baseline to Month 1^c^	30	1.27 (0.96–1.68)	.089	30	1.21 (0.78–1.89)	.39
N-terminal pro-B-type natriuretic peptide						
Baseline	43	0.96 (0.72–1.29)	.80	46	1.40 (1.06–1.85)	.017
Change from baseline to Month 1^c^	30	1.34 (.95–1.90)	.093	30	1.39 (1.02–1.89)	.036
High-sensitivity troponin T						
Baseline	43	1.22 (0.90–1.66)	.20	46	.98 (0.72–1.32)	.88
Change from baseline to Month 1^c^	30	1.91 (1.17–3.11)	.010	30	1.17 (0.72–1.91)	.52
C-reactive protein						
Baseline	43	1.14 (0.86–1.52)	.37	46	1.44 (1.13–1.83)	.004
Change from baseline to Month 1^c^	30	1.16 (0.77–1.74)	.47	30	1.12 (0.71–1.77)	.63
Change from baseline to Month 3^d^	14	1.88 (1.09–3.24)	.023	16	.62 (0.32–1.21)	.16

^a^All baseline, Month 1, and Month 3 predictors were log transformed.

^b^Per 1-unit standard deviation increase.

^c^Adjusted for baseline values.

^d^Adjusted for baseline values and change from baseline to Month 1.

**Table 3:** Adjusted association between biomarkers and risk of venous thromboembolism and clinically relevant bleeding in cancer patients from the AVERT trial (adjusted for age and advanced/metastatic cancer)

Predictor^a^	Venous thromboembolism			Bleeding		
	*N* of events	Subdistribution hazard ratio (95% confidence interval)^b^	*P*-value	*N* of events	Subdistribution hazard ratio (95% confidence interval)^b^	*P*-value
Growth differentiation factor-15						
Baseline	43	1.36 (1.01–1.84)	.045	46	1.09 (0.78–1.51)	.62
Change from baseline to Month 1^c^	30	1.27 (0.95–1.68)	.10	30	1.23 (0.79–1.91)	.37
N-terminal pro-B-type natriuretic peptide						
Baseline	43	0.94 (0.70–1.27)	.70	46	1.44 (1.08–1.92)	.014
Change from baseline to Month 1^c^	30	1.30 (0.89–1.89)	.18	30	1.41 (1.04–1.91)	.027
High-sensitivity troponin T						
Baseline	43	1.22 (0.88–1.69)	.23	46	.98 (0.71–1.36)	.92
Change from baseline to Month 1^c^	30	1.89 (1.14–3.16)	.014	30	1.15 (0.76–1.74)	.51
C-reactive protein						
Baseline	43	1.22 (0.91–1.64)	.18	46	1.38 (1.07–1.76)	.012
Change from baseline to Month 1^c^	30	1.12 (0.74–1.69)	.58	30	1.13 (0.72–1.78)	.60

^a^All baseline, Month 1, and Month 3 predictors were log transformed.

^b^Per 1-unit standard deviation increase.

^c^Adjusted for baseline values.

**Table 4:** Unadjusted association between biomarkers and bleeding in the apixaban group

Predictor^a^	*N*	Bleeding		
		*N* of events	Subdistribution hazard ratio (95% confidence interval)^b^	*P*-value
Growth differentiation factor-15				
Baseline	258	30	1.15 (0.80–1.66)	.44
Change from baseline to Month 1^c^	217	17	1.37 (0.69–2.72)	.37
N-terminal pro-B-type natriuretic peptide				
Baseline	258	30	1.58 (1.15–2.18)	.005
Change from baseline to Month 1^c^	217	17	1.17 (0.79–1.75)	.42
High-sensitivity troponin T				
Baseline	258	30	1.12 (0.81–1.56)	.50
Change from baseline to Month 1^c^	217	17	1.26 (0.65–2.45)	.42
C-reactive protein				
Baseline	258	30	1.46 (1.13–1.89)	.004
Change from baseline to Month 1^c^	217	18	1.03 (0.59–1.80)	.92
Change from baseline to Month 3^d^	181	11	0.66 (.30–1.47)	.31

^a^All predictors were log transformed except for the changes from baseline to Month 1.

^b^Per 1-unit standard deviation increase.

^c^Adjusted for baseline values.

^d^Adjusted for baseline values and change from baseline to Month 1.

In our analyses of changes over time, we again found no evidence of non-linear associations, permitting us to fit Fine and Gray competing risk regression models without restricted cubic splines. According to our multivariable analyses, increasing hs-TnT levels from baseline to Month 1 (i.e. log hs-TnT at Month 1 minus log hs-TnT at baseline) was associated with an increased risk of VTE [SHR 1.89 (95% CI 1.14–3.16)] but not bleeding (*Table 3*). This association was consistent in sensitivity analyses without imputations, adjusting for non-chemotherapy cancer therapies, adjusting for apixaban, and in patients receiving placebo (see Supplementary data online, *Tables S1*–*S5*). Although we did not perform multivariable analyses given the lower number of events and sample size, an increase in C-reactive protein levels from baseline to Month 3 was also associated with VTE risk [SHR 1.89 (95% CI 1.14–3.16)] in the univariate model but not with bleeding (*Table 2*). When C-reactive protein was analysed categorically in subgroups of C-reactive protein change (i.e. C-reactive protein rise and C-reactive protein decline),^30^ patients with a C-reactive protein decline of 50% or greater in the first 3 months had a lower risk of VTE compared with those without [SHR 0.33 (95% CI 0.11–1.02)] (see Supplementary data online, *Table S6*). Additionally, our analyses revealed that increasing NT-proBNP levels from baseline to Month 1 was associated with an increased risk of bleeding [SHR 1.41 (95% CI 1.04–1.91); *Table 3*]. However, a high hazard ratio was also reported in relation to VTE, and in our subgroup analysis of patients randomized to apixaban, the hazard ratio was attenuated (*Table 4*). Moreover, a sensitivity multiplicative interaction analysis suggested that apixaban may modify the relationship between NT-proBNP change and clinical outcomes, potentially amplifying the association between NT-proBNP change with VTE risk, while a possible attenuation of bleeding risk was observed, though not statistically significant (see Supplementary data online, *Table S7*).

**Figure 2:**
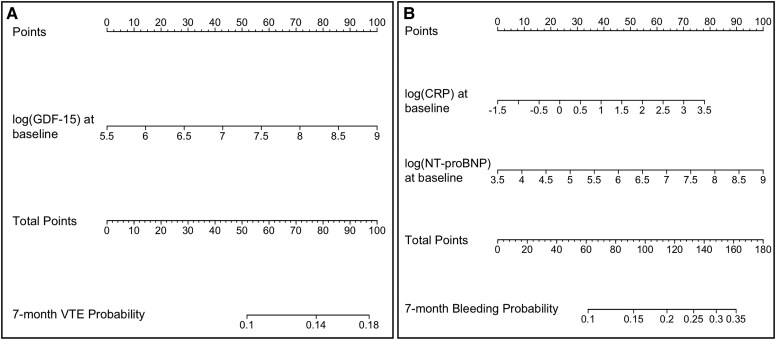
Nomograms for predicting the risk of (*A*) venous thromboembolism and (*B*) clinically relevant bleeding using baseline biomarkers

**Figure 3:**
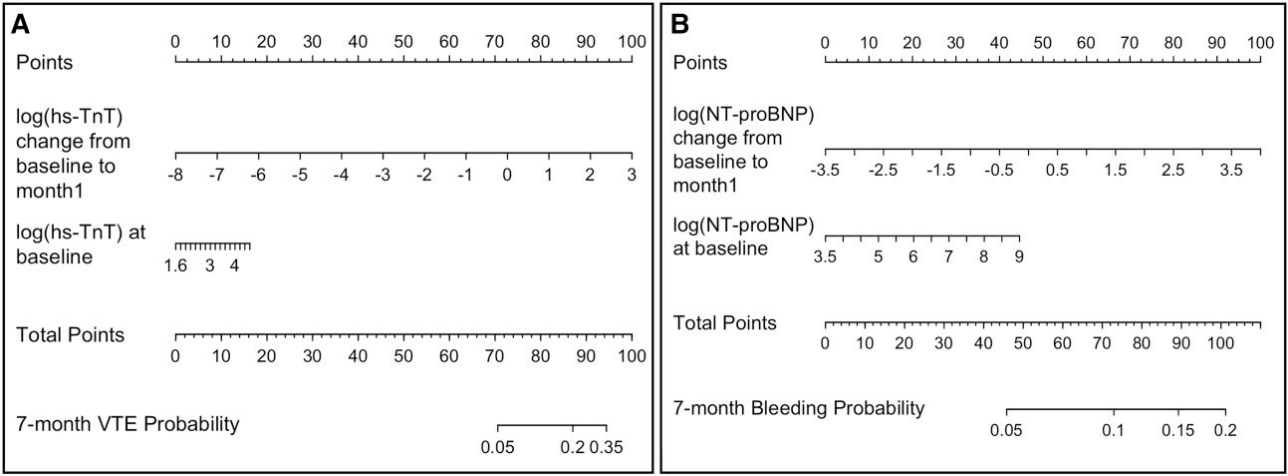
Nomograms for predicting the risk of (*A*) venous thromboembolism and (*B*) clinically relevant bleeding using biomarker levels at baseline and Month 1

Four nomograms were generated based on the results of our biomarker analyses—one for predicting the risk of VTE using baseline GDF-15, one for predicting the risk of bleeding using baseline biomarkers (i.e. C-reactive protein and NT-proBNP), one for predicting the risk of VTE using changes in hs-TnT levels from baseline to Month 1, and one for predicting the risk of bleeding using changes in NT-proBNP levels from baseline to Month 1 (*Figures 2* and *3*). In all four nomograms, the number of assigned points and predicted risk of outcome increased with increasing levels of biomarkers. To illustrate how these nomograms work, we consider a hypothetical example for predicting the risk of bleeding using baseline biomarkers (*Figure 2*). If a patient has a natural log C-reactive protein of 1 (equivalent to 2.7 mg/dL) and a natural log NT-proBNP of 7 (equivalent to 1100 pg/mL), they would be assigned 50 and 40 points, respectively, based on the top point axis. The sum of these values would then be used to generate a predicted risk probability using the bottom point axis. In this example, the patient would have a total of 90 points, which would correspond to an estimated 15% risk of bleeding. Based on our baseline risk biomarker-based nomograms, patients with a predicted VTE risk of ≥10% and a predicted bleeding risk of <15% may benefit from thromboprophylaxis (see Supplementary data online, *Table S8*). Alternative thresholds were explored (data not shown), but the selected predicted probability thresholds provided the best balance between clinical relevance and reliable stratification.

In our analyses evaluating the incremental value of biomarkers added to existing clinical risk tools, for VTE risk, adding GDF-15 to the Khorana, Protecht, and Vienna CATS scores improved both the area under the curve (AUC) and the C-statistic across all models (see Supplementary data online, *Table S9*). In a stratified analysis by the Khorana score, elevated GDF-15 levels were associated with increased VTE risk among patients with a Khorana score of 2 [*n* = 338, SHR 1.44 (95% CI 0.99–2.08); Supplementary data online, *Table S10*], whereas no significant association was observed among those with a Khorana score of 3 or more [*n* = 176, SHR 1.10 (95% CI 0.71–1.72), *P* = .66; Supplementary data online, *Table S10*]. For clinically relevant bleeding, we evaluated whether NT-proBNP and C-reactive protein provided additional predictive value beyond the CAT-bleed score. Incorporating these biomarkers improved model performance, increasing the AUC from 0.55 to 0.64 and the C-statistic from 0.55 to 0.66 (see Supplementary data online, *Table S9*).

## Discussion

In this *post hoc* analysis of the AVERT trial involving cancer patients at intermediate to high risk of VTE (Khorana score of ≥2), higher baseline GDF-15 was associated with an increased risk of VTE but not bleeding, with this association being more pronounced in patients with a Khorana score of 2 (as compared with a higher Khorana score). In contrast, higher baseline C-reactive protein and NT-proBNP levels were associated with an increased risk of bleeding, but not VTE, during 7 months of follow-up after chemotherapy initiation. Our results also suggested that an increase in hs-TnT levels from baseline to Month 1 and an increase in C-reactive protein levels from baseline to Month 3 were associated with an increased 7-month risk of VTE but not bleeding. Lastly, we found that increasing NT-proBNP levels from baseline to Month 1 was associated with an increased risk of bleeding, though this association was no longer significant in patients randomized to apixaban. Based on these results, four nomograms were generated for the prediction of VTE and bleeding in patients with cancer, and we confirmed that adding these biomarkers to existing risk tools improved their predictive performance (*Structured Graphical Abstract*).

Our findings suggest that at baseline, biomarkers such as GDF-15, C-reactive protein, and NT-proBNP may help optimize risk assessment for VTE or bleeding in patients with cancer starting chemotherapy, potentially facilitating decision-making regarding primary thromboprophylaxis. In particular, GDF-15, a stress-related cytokine associated with inflammation, showed potential as a baseline predictor for VTE with no link to bleeding, though this contrasts with results from the Vienna CATS studies, which reported a relationship between GDF-15 and bleeding risk with no link to VTE.^15,16^ The differences in study patient populations and follow-up durations between the AVERT trial and the Vienna CATS cohort may explain these inconsistencies. Particularly, the AVERT trial included patients at intermediate to high risk of VTE (Khorana score of ≥2) with almost half of the patients randomized to receive apixaban as primary thromboprophylaxis and followed for 7 months, whereas the Vienna CATS cohort included a broader cancer population that were followed up to 24 months, and prophylactic anticoagulation was not routinely given in their cohort. Thus, other studies are needed to determine the role of baseline GDF-15 in cancer patients.

Additionally, in our study, we found an association between C-reactive protein, another inflammatory-related biomarker, and an increased risk of bleeding. Although GDF-15 and C-reactive protein are both inflammatory-related biomarkers, they have different properties, which may explain why one is associated with VTE while the other is associated bleeding. Growth differentiation factor-15 is a stress-response cytokine expressed in response to inflammation, oxidative stress, hypoxia, telomere erosion, and oncogene activation,^36^ whereas C-reactive protein is an acute-phase reactant protein expressed primarily in response to infection and inflammation.^37^ Growth differentiation factor-15 is thus known to have a broader role in inflammatory or stress responses compared with C-reactive protein. A possible mechanism linking GDF-15 to VTE is via the activation of the Smad2/pSmad2/Snail pathway by GDF-15 expression, which promotes endothelial-to-mesenchymal transition and reduces their antithrombotic ability.^38^ The mechanistic link between C-reactive protein and bleeding is not fully understood, but C-reactive protein could be an indicator of other comorbidities associated with increased risk of bleeding such as atrial fibrillation^39^ or hypertension.^40^

Baseline NT-proBNP was also identified as a predictor for bleeding. N-terminal pro-B-type natriuretic peptide is a cardiac marker secreted in response to myocyte stretch, hypoxia, and endocrine activation.^22,41^ While it is a biomarker of cardiac stress primarily used to monitor heart failure patients,^42^ our findings suggest that NT-proBNP may also be useful in cancer patients initiating chemotherapy to predict bleeding. The potential link between NT-proBNP and bleeding could be related to the physiological effects of its biologically active counterpart, B-type natriuretic peptide (BNP), which inhibits the renin–angiotensin system and the sympathetic nervous system,^22^ potentially influencing fibrinolysis and haemostasis. Moreover, elevated NT-proBNP has been linked to several bleeding risk factors including low haemoglobin,^43,44^ hypertension,^45^ metastatic disease,^46^ and systematic inflammation,^47^ which could also explain the increased susceptibility to bleeding in patients with elevated NT-proBNP levels.

Beyond baseline measures, longitudinal changes in biomarkers can provide insights into changes in risk during treatment (i.e. chemotherapy or anticoagulation therapy). Our findings suggest that increasing hs-TnT levels from baseline to Month 1 and increasing C-reactive protein levels from baseline to Month 3 are associated with higher VTE risk. Elevated 1-month hs-TnT (>14 pg/mL) was previously identified as predicting future VTE events,^13^ and our study extends this by demonstrating that increases in hs-TnT levels from baseline to Month 1 may serve as an indicator of increased VTE risk after initiating chemotherapy. Since hs-TnT is a marker of myocardial damage, rising hs-TnT may be related to the damaging effects of chemotherapy on the cardiovascular system.^48^

Additionally, the positive association between C-reactive protein changes at 3 months and VTE aligns with previous research. A study by Moik *et al.*^30^ found that an early C-reactive protein rise (defined as a two-fold or greater increase in C-reactive protein levels in the first 3 months from baseline) was associated with increased VTE risk in cancer patients treated with immune checkpoint inhibitors.^30^ Since our study included cancer patients initiating any chemotherapy (not limited to immune checkpoint inhibitors), our findings suggest that this relationship may also extend to a broader cancer population, though confirmation in larger studies is needed due to our small sample size and low number of events. However, in a study of patients with metastatic colorectal cancer, patients who developed thrombosis tended to have increasing high-sensitivity C-reactive protein levels during the course of treatment after 2 months of chemotherapy,^49^ aligning with our results. Thus, there may be an inflammatory response occurring during the first few months of chemotherapy, increasing the likelihood of thrombosis. The lack of 1-month C-reactive protein–VTE association in our study raises the possibility that a shorter follow-up period may not be sufficient to capture the inflammatory effects of chemotherapy, supporting future research on C-reactive protein changes at 2–3 months.

Finally, increasing NT-proBNP levels from baseline to Month 1 was associated with increased bleeding risk, but an elevated hazard ratio was observed for VTE contraindicating its use as a potential bleeding predictor. Additionally, in patients randomized to apixaban, a lower hazard ratio was noted, suggesting that it may not be a useful predictor for bleeding complications in patients on anticoagulation. The reduced effect measure in apixaban patients could be due the pleiotropic effects of direct oral anticoagulants (DOACs), which according to Mele *et al.*^50^ may lower NT-proBNP through their anti-inflammatory and endothelial modulating actions. Therefore, NT-proBNP may not be a useful longitudinal biomarker to use for monitoring changes in risk of bleeding in cancer patients on DOACs.

The current study has multiple clinical implications. For one, this study highlights the role of two inflammatory-related markers and two cardiac markers for assessing, as well as monitoring, the risks of VTE and bleeding in patients with cancer starting chemotherapy. The associations identified in this study could help to further refine risk assessment models such as the Khorana score to guide decisions about thromboprophylaxis. Although these biomarkers are not routinely measured, some guidelines recommend assessing some of these biomarkers for other cancer-related outcomes including cardiotoxicity and cancer-related fatigue.^51–53^ Therefore, these biomarkers may increasingly become part of routine clinical practice, especially with the rapid advancements in technology. Moreover, in this study, we generated four nomograms to predict individual risk of VTE and bleeding based on the significant biomarkers identified in this study. These nomograms may help to better stratify patients’ risk, tailor treatment strategies, and monitor responses to therapy more effectively. As these nomograms are refined and validated, they could potentially become a helpful tool to make decisions about thromboprophylaxis and bleeding management, leading to improved patient outcomes. Our analyses also revealed that the addition of biomarkers to existing clinical risk models improved their ability to predict outcomes.

Our study has limitations that should be considered. First, although we found significant associations between select biomarkers and future VTE or bleeding risks, we cannot definitively establish causality. Second, the modest sample size and number of events limited our ability to control for all potential confounders; therefore, residual confounding may be present. Particularly, some biomarkers may be influenced by comorbidities or therapies (e.g. surgeries and radiation therapy) common in cancer patients. However, we adjusted for age and advanced cancer diagnosis since these are well-known time-invariant risk factors for biomarker variability and VTE and bleeding risk. Additionally, we performed sensitivity analyses adjusting for targeted therapy and radiation therapy to assess the robustness of the results, which did not alter the observed associations. No surgical procedures occurred at the time of biomarker measurement, minimizing the potential confounding effect of peri-operative changes in biomarker levels. In contrast, the absence of detailed comorbidity data limited our ability to evaluate the independent contribution of these conditions to biomarker variability and observed associations. Third, the AVERT trial included only selected cancer patients with intermediate to high VTE risk initiating chemotherapy defined by a Khorana score of ≥2. Additionally, some cancer types were excluded, so the results may not be generalizable to all cancer types (e.g. acute leukaemia and myeloproliferative neoplasms). Similarly, since the biomarkers were measured at baseline, Month 1, and Month 3 after initiating chemotherapy, the predictive ability of the biomarkers may not extend to other timepoints and in cancer patients initiating other therapies. Investigating a different length of follow-up may be of interest to other researchers; however, most VTE events occur within the first 6 months after diagnosis,^54^ and most bleeding events likely occur while on anticoagulation. It is also important to note that the biomarkers, at different timepoints, were measured in different labs, which may have led to variations in biomarker values. Additionally, hazard ratios reflect average effects over the study period, and period-specific estimates may be subject to selection bias, although SHRs remain appropriate for describing associations in this study. Lastly, this study does not address the practicality and cost-effectiveness of implementing the use of these biomarkers in clinical practice.

In conclusion, our study showed that elevated baseline GDF-15 was associated with increased VTE risk, while elevated baseline NT-proBNP and C-reactive protein levels were associated with a higher bleeding risk in cancer patients at intermediate to high risk of VTE (Khorana score of ≥2). Changes in hs-TnT and C-reactive protein over time could also predict VTE risk. Four nomograms were generated based on these findings, which may serve as a valuable tool for risk assessment and monitoring. Further large prospective studies are needed to confirm our findings and the clinical utility of these biomarkers.

## Supplementary Material

ehaf1002_Supplementary_Data

## Declarations

### Data Availability

The data supporting the findings of this study are available from the AVERT trial primary investigators upon reasonable request.

### Pre-registered Clinical Trial Number

The pre-registered clinical trial number for the AVERT trial is NCT02048865.
