# Supplementary material for: Venous thromboembolism and bleeding in cancer patients: role of inflammatory and cardiac biomarkers
Source: Eur Heart J. 2025 Dec 12;47(16):1933–45. doi: 10.1093/eurheartj/ehaf1002 (PMC13099213; doi:10.1093/eurheartj/ehaf1002)
Supplement: ehaf1002_Supplementary_Data [file ehaf1002_supplementary_data.docx]

**Supplementary Table 1. Sensitivity analysis without imputation (adjusted for age and advanced/metastatic cancer)**

| **Predictor^a^** | **N** | **VTE** | | | **N** | **Bleeding** | | |
| --- | --- | --- | --- | --- | --- | --- | --- | --- |
|  |  | **N of events** | **SHR (95% CI)^d^** | ***p-value*** |  | **N of events** | **SHR (95% CI)^d^** | ***p-value*** |
| **GDF-15** |  |  |  |  |  |  |  |  |
| **Baseline** | 445 | 35 | 1.46 (1.05-2.04) | *0.026* | 445 | 39 | 1.08 (0.75-1.54) | *0.69* |
| **Change from baseline to month-1^b^** | 393 | 25 | 1.36 (0.99-1.87) | *0.055* | 387 | 26 | 1.38 (0.90-2.12) | *0.14* |
| **NT-ProBNP** |  |  |  |  |  |  |  |  |
| **Baseline** | 442 | 33 | 1.04 (0.74-1.47) | *0.81* | 442 | 39 | 1.43 (1.02-2.00) | *0.037* |
| **Change from baseline to month-1^b^** | 391 | 23 | 1.60 (0.98-2.61) | *0.060* | 385 | 25 | 1.64 (1.13-2.38) | *0.009* |
| **hs-TnT** |  |  |  |  |  |  |  |  |
| **Baseline** | 419 | 33 | 1.30 (0.92-1.84) | *0.14* | 419 | 39 | 0.95 (0.66-1.37) | *0.79* |
| **Change from baseline to month-1^b^** | 372 | 23 | 1.92 (1.11-3.30) | *0.019* | 366 | 25 | 1.20 (0.66-2.18) | *0.55* |
| **CRP** |  |  |  |  |  |  |  |  |
| **Baseline** | 456 | 37 | 1.29 (0.94-1.76) | *0.11* | 456 | 40 | 1.37 (1.07-1.75) | *0.012* |
| **Change from baseline to month-1^b^** | 403 | 25 | 1.19 (0.78-1.81) | *0.41* | 396 | 26 | 1.17 (0.69-1.98) | *0.55* |
| **Unadjusted change from baseline to month-3^c^** | 354 | 14 | 2.03 (1.14-3.60) | *0.016* | 342 | 16 | 0.64 (0.32-1.28) | *0.21* |

**Abbreviations:** body mass index (BMI), c-reactive protein (CRP), growth differentiation factor-15 (GDF-15), N-terminal pro-B-type natriuretic peptide (NT-proBNP), high-sensitivity troponin-T (hs-TnT), standard deviation (SD), subdistribution hazard ratio (SHR), venous thromboembolism (VTE)

^a^ all predictors were log transformed except for the changes from baseline to month-1

^b^ adjusted for baseline values

^c^ adjusted for baseline values and change from baseline to month-1

^d^ Per 1-unit standard deviation increase

**Supplementary Table 2. Unadjusted association between biomarkers and VTE in Placebo group**

| **Predictor^a^** | **N** | **VTE** | | |
| --- | --- | --- | --- | --- |
|  |  | **N of events** | **SHR (95% CI)^d^** | ***p-value*** |
| **GDF-15** |  |  |  |  |
| **Baseline** | 256 | 30 | 1.29 (0.95-1.76) | *0.10* |
| **Change from baseline to month-1^b^** | 231 | 23 | 1.22 (0.86-1.74) | *0.27* |
| **NT-ProBNP** |  |  |  |  |
| **Baseline** | 256 | 30 | 1.01 (0.70-1.45) | *0.97* |
| **Change from baseline to month-1^b^** | 231 | 23 | 1.06 (0.72-1.56) | *0.78* |
| **hs-TnT** |  |  |  |  |
| **Baseline** | 256 | 30 | 1.27 (0.91-1.77) | *0.16* |
| **Change from baseline to month-1^b^** | 231 | 23 | 2.71 (1.27-5.81) | *0.010* |
| **CRP** |  |  |  |  |
| **Baseline** | 256 | 30 | 1.13 (0.82-1.57) | *0.46* |
| **Change from baseline to month-1^b^** | 231 | 23 | 1.27 (0.77-2.10) | *0.34* |
| **Change from baseline to month-3^c^** | 190 | 12 | 2.02 (1.09-3.76) | *0.026* |

**Abbreviations:** body mass index (BMI), c-reactive protein (CRP), growth differentiation factor-15 (GDF-15), N-terminal pro-B-type natriuretic peptide (NT-proBNP), high-sensitivity troponin-T (hs-TnT), standard deviation (SD), subdistribution hazard ratio (SHR), venous thromboembolism (VTE)

^a^ all predictors were log transformed except for the changes from baseline to month-1

^b^ adjusted for baseline values

^c^ adjusted for baseline values and change from baseline to month-1

^d^ Per 1-unit standard deviation increase

**Supplementary Table 3. Unadjusted association between biomarkers and VTE in Apixaban group**

| **Predictor^a^** | **N** | **VTE** | | |
| --- | --- | --- | --- | --- |
|  |  | **N of events** | **SHR (95% CI)^c^** | ***p-value*** |
| **GDF-15** |  |  |  |  |
| **Baseline** | 258 | 13 | 1.40 (0.76-2.59) | *0.28* |
| **Change from baseline to month-1^b^** | 223 | 7 | 1.41 (0.93-2.15) | *0.11* |
| **NT-ProBNP** |  |  |  |  |
| **Baseline** | 258 | 13 | 0.92 (0.57-1.48) | *0.73* |
| **Change from baseline to month-1^b^** | 223 | 7 | 2.42 (1.36-4.30) | *0.003* |
| **hs-TnT** |  |  |  |  |
| **Baseline** | 258 | 13 | 1.07 (0.54-2.11) | *0.85* |
| **Change from baseline to month-1^b^** | 223 | 7 | 1.17 (0.61-2.23) | *0.63* |
| **CRP** |  |  |  |  |
| **Baseline** | 258 | 13 | 1.18 (0.67-2.08) | *0.57* |
| **Change from baseline to month-1^b^** | 223 | 7 | 0.79 (0.53-1.16) | *0.22* |

**Abbreviations:** body mass index (BMI), c-reactive protein (CRP), growth differentiation factor-15 (GDF-15), N-terminal pro-B-type natriuretic peptide (NT-proBNP), high-sensitivity troponin-T (hs-TnT), standard deviation (SD), subdistribution hazard ratio (SHR), venous thromboembolism (VTE)

^a^ all predictors were log transformed except for the changes from baseline to month-1

^b^ adjusted for baseline values

^c^ Per 1-unit standard deviation increase

**Supplementary Table 4. Adjusted association between biomarkers and risk of venous thromboembolism (VTE) and clinically relevant bleeding in cancer patients from the AVERT trial (adjusted for non-chemotherapy cancer therapies occurring at the time of biomarker measurements)**

| **Predictor^a^** | **VTE** | | | **Bleeding** | | |
| --- | --- | --- | --- | --- | --- | --- |
|  | **N of events** | **SHR (95% CI)^d^** | ***p-value*** | **N of events** | **SHR (95% CI)^d^** | ***p-value*** |
| **GDF-15** |  |  |  |  |  |  |
| **Baseline ^b^** | 43 | 1.31 (0.99-1.73) | *0.058* | 46 | 1.10 (0.79-1.52) | *0.58* |
| **Change from baseline to month-1^c^** | 30 | 1.26 (0.96-1.65) | *0.10* | 30 | 1.18 (0.77-1.81) | *0.45* |
| **NT-ProBNP** |  |  |  |  |  |  |
| **Baseline ^b^** | 43 | 1.06 (0.78-1.44) | *0.71* | 46 | 1.40 (1.06-1.85) | *0.016* |
| **Change from baseline to month-1^c^** | 30 | 1.32 (0.92-1.90) | *0.13* | 30 | 1.35 (0.97-1.97) | *0.071* |
| **hs-TnT** |  |  |  |  |  |  |
| **Baseline ^b^** | 43 | 1.28 (0.96-1.71) | *0.10* | 46 | 0.98 (0.72-1.32) | *0.88* |
| **Change from baseline to month-1^c^** | 30 | 1.92 (1.20-3.08) | *0.007* | 30 | 1.19 (0.71-2.00) | *0.50* |
| **CRP** |  |  |  |  |  |  |
| **Baseline ^b^** | 43 | 1.20 (0.90-1.62) | *0.22* | 46 | 1.42 (1.12-1.81) | *0.004* |
| **Change from baseline to month-1^c^** | 30 | 1.10 (0.71-1.70) | *0.66* | 30 | 1.03 (0.62-1.71) | *0.91* |

**Abbreviations:** body mass index (BMI), c-reactive protein (CRP), growth differentiation factor-15 (GDF-15), N-terminal pro-B-type natriuretic peptide (NT-proBNP), high-sensitivity troponin-T (hs-TnT), standard deviation (SD), subdistribution hazard ratio (SHR), venous thromboembolism (VTE)

^a^ all baseline and month-1 predictors were log transformed

^b^ adjusted for targeted and radiation therapy at the time of baseline blood sampling

^c^ adjusted for baseline values and exposure to targeted or radiation therapy occurring during the first month before month-1 sampling

^d^ Per 1-unit standard deviation increase

**Supplementary Table 5. Adjusted association between biomarkers and risk of venous thromboembolism (VTE) and clinically relevant bleeding in cancer patients from the AVERT trial (adjusted for apixaban)**

| **Predictor^a^** | **VTE** | | | **Bleeding** | | |
| --- | --- | --- | --- | --- | --- | --- |
|  | **N of events** | **SHR (95% CI)** | ***p-value*** | **N of events** | **SHR (95% CI)** | ***p-value*** |
| **GDF-15** |  |  |  |  |  |  |
| **Baseline ^b^** | 43 | 1.32 (1.00-1.74) | *0.053* | 46 | 1.08 (0.78-1.49) | *0.64* |
| **Change from baseline to month-1^b^** | 30 | 1.28 (0.95-1.71) | *0.10* | 30 | 1.22 (0.79-1.88) | *0.38* |
| **NT-ProBNP** |  |  |  |  |  |  |
| **Baseline ^b^** | 43 | 0.98 (0.73-1.31) | *0.88* | 46 | 1.39 (1.05-1.83) | *0.020* |
| **Change from baseline to month-1^b^** | 30 | 1.29 (0.90-1.85) | *0.17* | 30 | 1.40 (1.04-1.90) | *0.029* |
| **hs-TnT** |  |  |  |  |  |  |
| **Baseline ^b^** | 43 | 1.20 (0.90-1.61) | *0.21* | 46 | 0.98 (0.72-1.34) | *0.91* |
| **Change from baseline to month-1^b^** | 30 | 1.93 (1.15-3.26) | *0.013* | 30 | 1.18 (0.72-1.91) | *0.51* |
| **CRP** |  |  |  |  |  |  |
| **Baseline ^b^** | 43 | 1.14 (0.86-1.52) | *0.35* | 46 | 1.45 (1.13-1.86) | *0.004* |
| **Change from baseline to month-^b^** | 30 | 1.14 (0.76-1.72) | *0.53* | 30 | 1.13 (0.72-1.77) | *0.60* |

**Abbreviations:** body mass index (BMI), c-reactive protein (CRP), growth differentiation factor-15 (GDF-15), N-terminal pro-B-type natriuretic peptide (NT-proBNP), high-sensitivity troponin-T (hs-TnT), standard deviation (SD), subdistribution hazard ratio (SHR), venous thromboembolism (VTE)

^a^ all baseline and month-1 predictors were log transformed

^b^ Per 1-unit standard deviation increase

**Supplementary Table 6. Association between CRP change subgroups and risk of VTE and clinically relevant bleeding**

| **Predictor^a^** | **Model** | **VTE** | | | **Bleeding** | | |
| --- | --- | --- | --- | --- | --- | --- | --- |
|  |  | **N of events** | **SHR (95% CI)^d^** | ***p-value*** | **N of events** | **SHR (95% CI)^d^** | ***p-value*** |
| **CRP change subgroups*** | | | | | | | |
| **CRP rise** | Unadjusted | 14 | 1.27 (0.36-4.54) | *0.71* | 16 | 1.02 (0.30-3.55) | *0.97* |
|  | Adjusted | 14 | 1.13 (0.28-4.48) | *0.86* | 16 | 1.17 (0.33-4.14) | *0.81* |
| **CRP decline** | Unadjusted | 14 | 0.34 (0.12-0.96) | *0.042* | 16 | 1.43 (0.47-4.41) | *0.53* |
|  | Adjusted | 14 | 0.33 (0.11-1.02) | *0.054* | 16 | 1.30 (0.40-4.25) | *0.66* |

**Abbreviations:** body mass index (BMI), c-reactive protein (CRP), growth differentiation factor-15 (GDF-15), N-terminal pro-B-type natriuretic peptide (NT-proBNP), high-sensitivity troponin-T (hs-TnT), standard deviation (SD), subdistribution hazard ratio (SHR), venous thromboembolism (VTE)

***the CRP measurements at month-1 and month-3 were used to classify the subgroups; patients with a doubling or 50% decrease from baseline to month-1 OR month-3 were classified as CRP rise and CRP decline, respectively.**

**Supplementary Table 7. Interaction between apixaban use and biomarker levels in relation to VTE and clinically relevant bleeding risk in cancer patients from the AVERT trial**

| **Predictor^a^** | **VTE** | | | **Bleeding** | | |
| --- | --- | --- | --- | --- | --- | --- |
|  | **N of events** | **Interaction SHR (95% CI)** | ***p-value*** | **N of events** | **Interaction SHR (95% CI)** | ***p-value*** |
| **GDF-15** |  |  |  |  |  |  |
| **Baseline ^b^** | 43 | 1.11 (0.55-2.24) | *0.78* | 46 | 1.22 (0.60-2.50) | *0.58* |
| **Change from baseline to month-1^b^** | 30 | 1.14 (0.66-1.96) | *0.64* | 30 | 1.36 (0.59-3.09) | *0.47* |
| **NT-ProBNP** |  |  |  |  |  |  |
| **Baseline ^b^** | 43 | 0.91 (0.50-1.66) | *0.76* | 46 | 1.48 (0.77-2.85) | *0.24* |
| **Change from baseline to month-1^b^** | 30 | 2.24 (1.09-4.59) | *0.028* | 30 | 0.69 (0.39-1.22) | *0.20* |
| **hs-TnT** |  |  |  |  |  |  |
| **Baseline ^b^** | 43 | 0.86 (0.39-1.91) | *0.71* | 46 | 1.53 (0.67-3.49) | *0.31* |
| **Change from baseline to month-1^b^** | 30 | 0.50 (0.18-1.36) | *0.17* | 30 | 1.09 (0.38-3.09) | *0.87* |
| **CRP** |  |  |  |  |  |  |
| **Baseline ^b^** | 43 | 1.05 (0.55-2.03) | *0.88* | 46 | 1.40 (0.85-2.32) | *0.19* |
| **Change from baseline to month-^b^** | 30 | 0.62 (0.33-1.18) | *0.14* | 30 | 0.75 (0.29-1.91) | *0.54* |

**Abbreviations:** body mass index (BMI), c-reactive protein (CRP), growth differentiation factor-15 (GDF-15), N-terminal pro-B-type natriuretic peptide (NT-proBNP), high-sensitivity troponin-T (hs-TnT), standard deviation (SD), subdistribution hazard ratio (SHR), venous thromboembolism (VTE)

^a^ all baseline and month-1 predictors were log transformed

^b^ Per 1-unit standard deviation increase

**Supplementary Table 8. Observed rates of VTE and bleeding stratified by predicted risk groups based on baseline biomarker nomograms (Figure 2)**

| Risk Group* | VTE Rate | Bleeding Rate |
| --- | --- | --- |
| High VTE/Low Bleeding | 10.4% | 6.7% |
| High VTE/ High Bleeding | 7.3% | 22.0% |
| Low VTE/ High Bleeding | 4.8% | 14.3% |
| Low VTE/ Low Bleeding | 7.9% | 7.9% |

*High VTE risk group defined as predicted probability $\geq$10% and high bleeding risk group defined as predicted probability $\geq$15%

**Supplementary Table 9. Performance of existing risk models when adding predictive biomarkers**

| **Outcome** | **Model** | **AUC** | **C-statistic** |
| --- | --- | --- | --- |
| **VTE** | **Khorana Score only** | **0.59 (0.50-0.69)** | **0.58 (0.50-0.65)** |
|  | **Khorana Score + GDF-15** | **0.68 (0.57-0.78)** | **0.61 (0.53-0.70)** |
|  | **Protecht Score only** | **0.67 (0.58-0.76)** | **0.60 (0.53-0.68)** |
|  | **Protecht Score + GDF-15** | **0.74 (0.63-0.84)** | **0.64 (0.56-0.72)** |
|  | **ViennaCATS score only** | **0.61 (0.50-0.73)** | **0.59 (0.50-0.68)** |
|  | **ViennaCATS score + GDF-15** | **0.67 (0.55-0.77)** | **0.60 (0.52-0.70)** |
| **Clinically relevant bleeding** | **CAT-bleed linear predictor only** | **0.55 (0.44-0.67)** | **0.55 (0.46-0.64)** |
|  | **CAT-bleed linear predictor + NT-proBNP + CRP** | **0.64 (0.56-0.72)** | **0.66 (0.55-76)** |

**Abbreviations:** area under the curve (AUC), body mass index (BMI), c-reactive protein (CRP), growth differentiation factor-15 (GDF-15), N-terminal pro-B-type natriuretic peptide (NT-proBNP), high-sensitivity troponin-T (hs-TnT), venous thromboembolism (VTE)

**Supplementary Table 10. Stratified analysis of GDF-15 and VTE by Khorana Score**

| Khorana Score Group | SHR for VTE per 1 standard deviation increase of GDF-15 (95% Confidence intervals) | p-value |
| --- | --- | --- |
| Khorana Score = 2 | 1.44 (0.99-2.08) | 0.054 |
| Khorana Score $\geq$ 3 | 1.10 (0.71-1.72) | 0.66 |

**Abbreviations:** GDF-15=Growth differentiation factor-15, SHR=subdistribution hazard ratio, VTE=venous thromboembolism
